# Raja 42, a novel gamma lactam compound, is effective against *Clostridioides difficile*

**DOI:** 10.1371/journal.pone.0257143

**Published:** 2021-09-07

**Authors:** Alexis Fong, Megan Ross, Justin Boudreau, Reza Nokhbeh, Kim Tilbe, Hoyun Lee

**Affiliations:** 1 Health Sciences North Research Institute, Sudbury, Ontario, Canada; 2 Biomolecular Sciences Program, Laurentian University, Sudbury, Ontario, Canada; 3 Northern Ontario School of Medicine, Sudbury, Ontario, Canada; 4 Department of Medicine, the University of Ottawa Medical School, Ottawa, Ontario, Canada; United States Department of Agriculture, Agricultural Research Service, UNITED STATES

## Abstract

*Clostridioides difficile* infection (CDI) is the primary cause of hospital-acquired diarrhea, and responsible for over 500,000 enteric infections a year in the United States alone. Although most patients with CDI are successfully treated with metronidazole or vancomycin, the high rate of recurrence is still a serious problem, in which case these antibiotics are usually not very effective. The primary objective of this research is to develop a potentially effective therapeutic agent against *C*. *difficile* that are resistant to metronidazole or vancomycin. The susceptibility to metronidazole and vancomycin was examined with 194 *C*. *difficile* clinical isolates. Sixty of these isolates chosen based on a variety of criteria were examined for their susceptibility against the 4-chloro-1-piperidin-1ylmethyl-1H-indole-2,3-dione compound (Raja 42), a novel isatin–benzothiazole analogue containing a gamma-lactam structure, as we previously found that this novel compound is effective against a variety of different bacteria. Most of the 60 isolates were resistant to ceftriaxone and ciprofloxacin, raising the possibility that they might have been exposed previously to these or structurally similar antibiotics (e.g., β-lactam and quinolone compounds). Among the isolates, 48 (80%) and 54 (90%) were susceptible to metronidazole and vancomycin, respectively. Raja 42 was found to be effective against most of the isolates, especially so against metronidazole-resistant *C*. *difficile*. Most importantly, five isolates that show resistance to metronidazole and vancomycin were sensitive to Raja 42. Thus, Raja 42, a gamma lactam antibiotic, has the potential to effectively control *C*. *difficile* strains that are resistant to metronidazole and vancomycin.

## Introduction

*Clostridioides difficile*, a Gram-positive bacillus bacterium, is the primary cause of hospital-acquired diarrhea that often happens to patients with prior exposure to antimicrobial agents. It has been estimated that *C*. *difficile* infection (CDI) is responsible for over 500,000 enteric infections a year in the United States alone [1]. In addition to diarrhea, CDI also causes colitis, which can ultimately lead to death if left untreated [2]. Although most patients with CDI are successfully treated with metronidazole or vancomycin, the high rate of recurrence is still a serious problem [3,4]. The relapse is often associated with the emergence of antibiotic resistant bacterial strains. Therefore, the development of safe and powerful new drugs is highly desirable; unfortunately, however, the number of potentially effective novel antimicrobial agents in the pipeline is declining in recent years, raising serious concerns.

**Fig 1:**
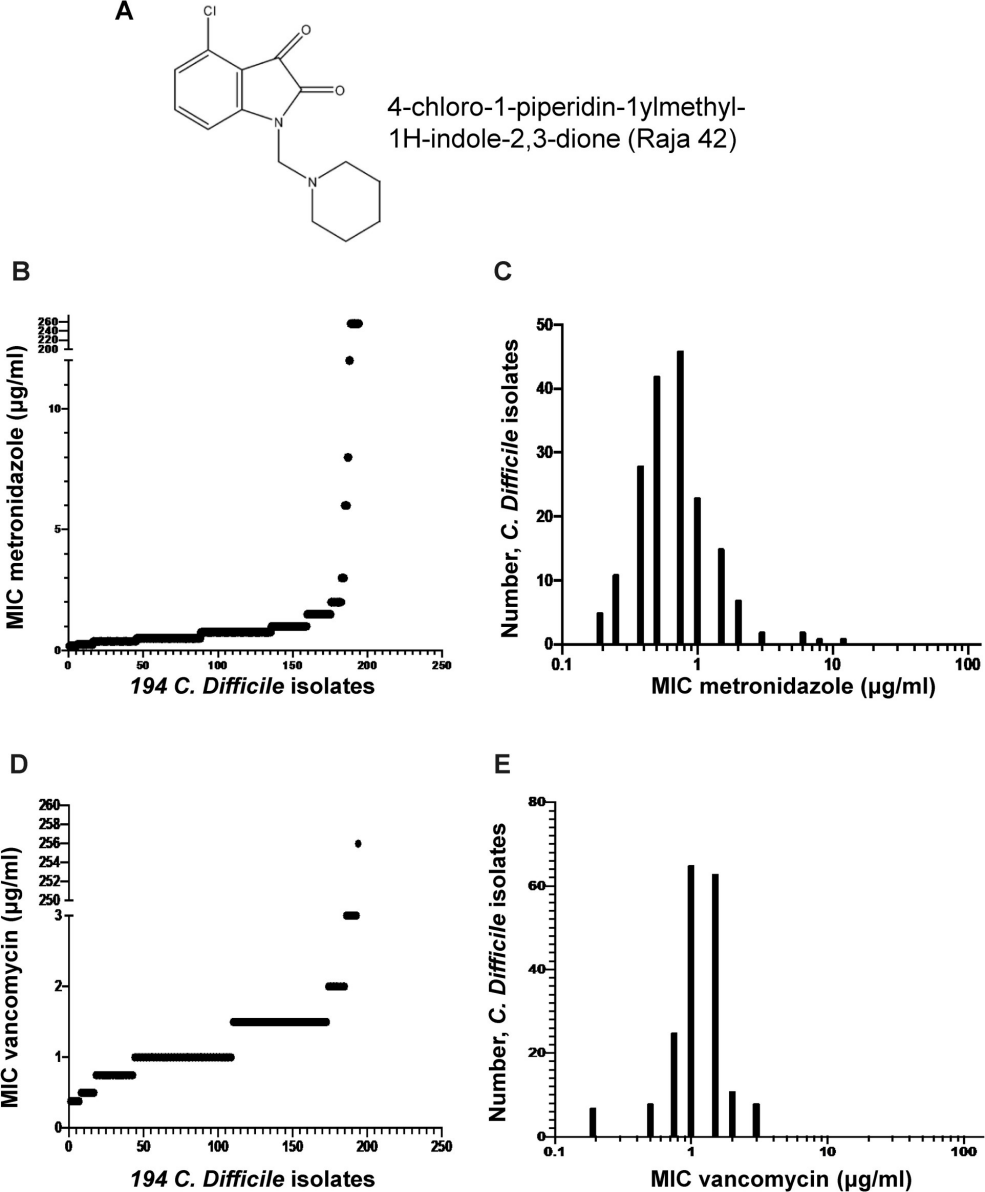
Distribution of metronidazole and vancomycin MIC against *C*. *difficile* clinical isolates. (A) The chemical structure and name of Raja 42 are shown. (B-E) Metronidazole and vancomycin were determined for their MIC values on 194 *C*. *difficile* clinical isolates (S1 Table). The susceptibility of these isolates to metronidazole or vancomycin was determined according to the guideline recommended by EUCAST and CLSI. The MIC value of metronidazole against *C*. *difficile* is 2 μg/mL, according to which 12 of the isolates were resistant to metronidazole (B, C). Vancomycin MIC against *C*. *difficile* is 2 μg/mL (D, E).

We previously synthesized and examined a series of isatin–benzothiazole analogs in an effort to develop an effective and safe anticancer drug, in which 4-chloro-1-piperidin-1ylmethyl-1H-indole-2,3-dione compound (Raja 42; Fig 1A) was included [5]. Raja 42 does not effectively kill normal human cells as its IC_50_ value is 93.72 μM, although it kills cancer cells better [5]. Since isatin-based compounds often show antimicrobial activities [6,7], we examined the aforementioned chemical library for their antimicrobial activities. We have found that Raja 42 is effective against both Gram-positive and Gram-negative bacteria, including *Escherichia coli*, methicillin-resistant *S*. *aureus* (MRSA), and *Helicobacter pylori* (manuscript in preparation). In this report, we describe the effects of Raja 42 against 60 *C*. *difficile* clinical isolates, which shows significant promise as a potential drug for the treatment of patients with CDI.

## Materials and methods

### Synthesis of Raja 42 (4-Chloro-1-piperidin-1-ylmethyl-1H-indole-2,3-dione)

The synthesis protocol of Raja 42 was described previously [5]. Briefly, to the solution of isatin or substituted isatin (0.9 g, 4.08 mmol) in 5 mL of absolute ethanol was added to a mixture of secondary amino compound (0.27 g, 1.35 mmol) and aqueous formaldehyde 37% (0.5 mL) also dissolved in 10 mL of absolute ethanol. The reaction mixture was stirred for 3 h at room temperature, and then refrigerated for 48 h to form crystals. The crystalline products were separated by filtration, washed with hexane, and vacuum dried. Recrystallization from ethanol rendered desired products in pure form. This compound was obtained as a dark brownish red solid in 75% yield.

### *C*. *difficile* clinical isolates and other bacterial strains

The 194 clinical *C*. *difficile* isolates collected from patients at the Health Sciences North hospital, Ontario, Canada was obtained from the Nokhbeh group of the Health Sciences North Research Institute. Quality Control and validation was carried out by comparing the minimum inhibitory concentration (MIC) of metronidazole and vancomycin against that of EUCAST published values for *C*. *difficile* ATCC 700057, *C*. *difficile* ATCC 9689, *C*. *difficile* ATCC 42596, *C*. *difficile* ATCC 43598 and *E*. *faecalis* ATCC 29212 (a negative control) strains. *C*. *difficile* cells were grown in Mueller-Hinton (MH) broth (catalog #CM0405, Oxoid Microbiology/Thermo Scientific, Nepean, Ontario, Canada), or Columbia Broth (CB) (catalog #CM0331, Oxoid Microbiology/Thermo Scientific) to allow sporulation. For active growth of *C*. *difficile*, the Brain Heart Infusion (BHI) broth (catalog #QB-48-0305, Nutri-Bact, Terrebonne, Quebec, Canada) was used. Bacteriological agar was supplemented with hemin (bovine origins, with >90% purity) (catalog #H9039-1G, Sigma-Aldrich, Oakville, Ontario, Canada), vitamin K1 (Sigma-Aldrich) and defibrinated sheep blood (Nutri-Bact), and then pre-reduced in the anaerobic chamber prior to inoculation with bacteria. The growth chamber was supplied with anaerobic gas mixture composed of 85% N_2_, 10% H_2_ and 5% CO_2_ to generate strict anaerobic atmosphere. The cultures were incubated at 36.8°C overnight or as needed.

### Antibiotic susceptibility test

The susceptibility test for *C*. *difficile* to metronidazole and vancomycin was carried out with Epsilometer testing (E-test) kits purchased from Biomérieux Canada (Saint-Laurent, Quebec, Canada). The E-test was performed according to the guideline published by the Clinical Laboratory Safety Institute (CLSI) and the European Committee on Antimicrobial Susceptibility Testing (EUCAST, 2019). Briefly, the test was carried out with a 100 mm round petri dish containing 4-mm thick Brucella agar supplemented with 5% laked sheep blood, 5 mg/L hemin, and 1 mg/L vitamin K1. The turbidity of over-night broth cultures was adjusted to one McFarland turbidity index by diluting the cultures with pre-reduced BHI broth prior to streaking with sterile cotton swabs on pre-reduced BBA plates. E-test strips were equilibrated to ambient temperature for 30 min before application onto the plates containing the swabbed cultures, followed by incubation in the inverted position under anaerobic conditions at 36.8°C for 24–30 h. Qualitative assessment was done by comparing the MIC of clinical isolates to that of quality control strains and published MIC values.

### *C*. *difficile* susceptibility test against Raja 42

Plate microdilution assay was used to determine the MIC values for *C*. *difficile* isolates using 96-well sterile plates as no commercially available test strips are yet available for this novel compound. MIC was determined using a 96-well plate and measuring turbidity at OD_600_ of treated samples against a blank sample. The *C*. *difficile* ATCC 9689 strain was initially used to optimize the protocol by finding the effective window of dilution range corresponding to 0.55 μg/mL to 600 μg/mL. The optimized protocol was applied to test the susceptibility of the 60 *C*. *difficile* clinical isolates to Raja 42, with the exception of the dilution range being appropriately modified to 0.55 μg/mL to 150 μg/mL. Bacterial culture was diluted to one McFarland turbidity prior to addition to 96-well plate. Each experiment was performed in triplicate unless specified otherwise.

### Statistical analysis

Values are expressed as mean ± standard error, and biological data was acquired through three independent experiments unless otherwise stated. Comparison between experimental groups was made by *p* value determination using one-way ANOVA or student t-test. The *p* value <0.05 is considered to be statistically significant. Analyses were performed using GraphPad Prism software, version 8.2.1 (San Diego, CA).

### Ethics

All the clinical isolates were anonymized prior to obtaining by us, and only collection months and years are shown to further obscure patient identities. Nevertheless, the Health Sciences North Research Ethical Board reviewed our application and approved it (Project #19–007).

## Results and discussion

### Examination of 194 *C*. *difficile* clinical isolates against metronidazole and vancomycin

We randomly chose 194 clinical isolates spanning collection time from June 2012 to March 2017 from the Nokhbeh *C*. *difficile* collection, and then determined their MICs/susceptibilities to metronidazole and vancomycin using E-test at the concentration range of 0.0016–256 μg/mL (S1 Table; data are summarised in Fig 1B–1E). As expected, all four *C*. *difficile* strains from ATCC (ATCC 9689, ATCC 43596, ATCC 43598, and ATCC 700057) were susceptible to both metronidazole and vancomycin, while *E*. *faecalis* (ATCC 29212) was sensitive to vancomycin but resistant to metronidazole. According to the EUCAST guideline, the antibiotic susceptibility breakpoints for *C*. *difficile* are defined sensitive when < 2 μg**/**mL and resistant when > 2 μg**/**mL. The resultant data determined by E-test for the 194 clinical isolates showed that 12 isolates (6.2%) and 9 isolates (4.6%) were resistant to metronidazole and vancomycin, respectively (Fig 1B–1E; S1 Table). It is noted that the resistant strains are mostly those isolated in 2014 and later years. The susceptibility profiles determined by MIC showed unimodal distribution for both metronidazole (Fig 1C) and vancomycin (Fig 1E), which are largely consistent with published *C*. *difficile* data by EUCAST (2019). The MIC_90_ of vancomycin was 2 μg**/**mL, which is consistent with the EUCAST guideline. Five isolates were resistant to vancomycin, one of which was resistant up to 256 μg**/**mL, the maximum dose used in this study.

### The novel Raja 42 γ-lactam compound is effective against *C*. *difficile*

**Fig 2:**
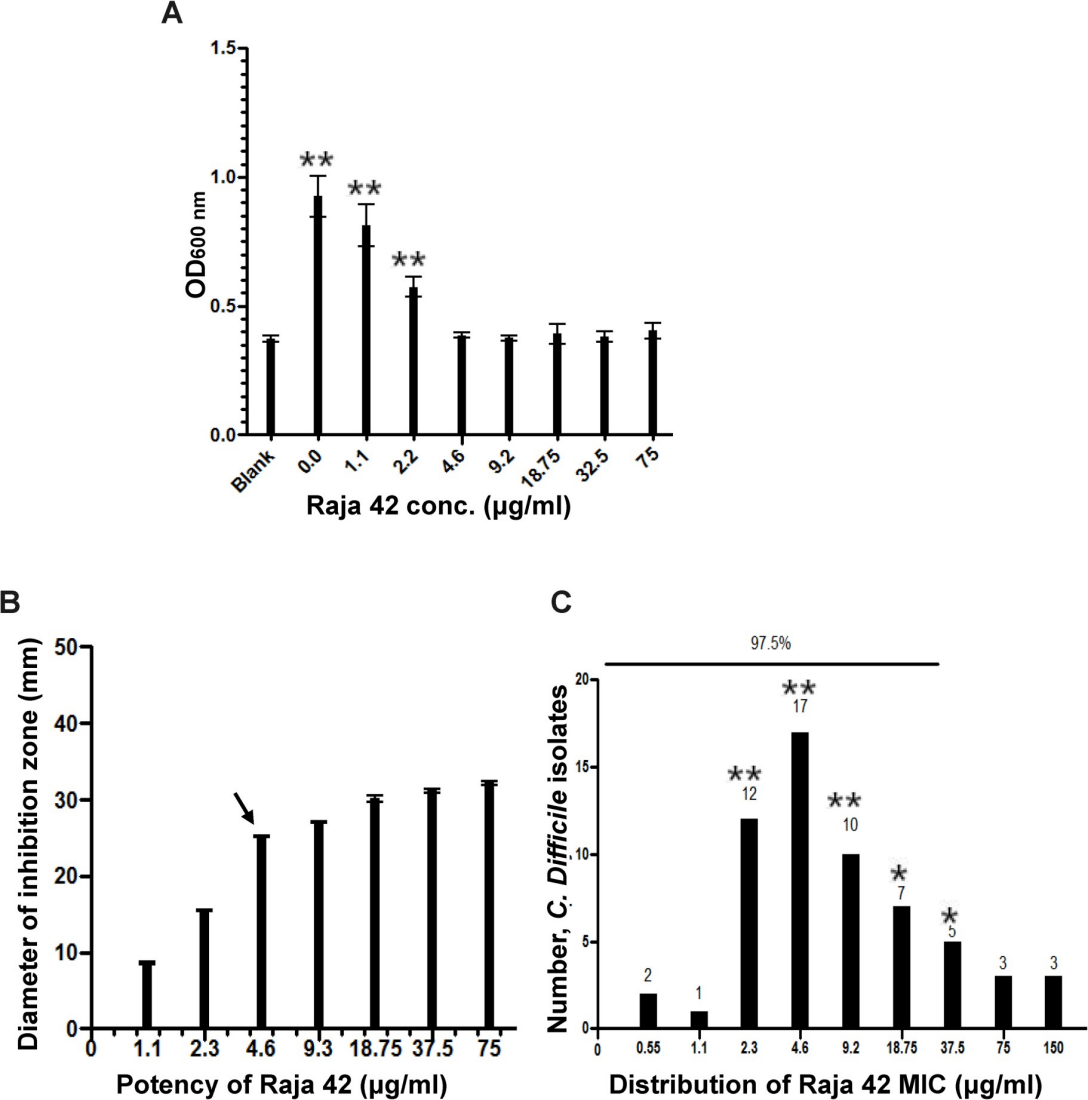
Distribution of Raja 42 MIC values against *C*. *difficile* isolates. (A) The average MIC measured using a 96-well format plotted for Raja 42 against *C*. *difficile* ATCC 9689 in triplicates. Note that due to the nature of the BHI medium used for culture, the broth was of a bright yellow colour and recorded as 0.5 turbidity when untreated. Similarly, the recording for treatments of 4.6 μg/mL-75 μg/mL displayed a similar turbidity of 0.5 allowing us to draw the conclusion that these were baseline levels in accordance to the untreated recording. The lowest concentration value (4.6 μg/mL) was stated as the MIC. (B) Determination of *C*. *difficile* ATCC 9689 MIC value using an agar plate dilution method. The MIC value of Raja 42 against ATCC 9689 *C*. *difficile* was determined to be 4.6 μg/mL (arrow). (C) The cut-off MIC value of Raja 42 against 60 *C*. *difficile* clinical isolates was 18.75 μg/mL. The isolates were treated with serial dilutions of Raja 42 ranging from 0.55 μg/mL to 150 μg/mL using 96-well plates. According to the EUCAST Epidemiological Cut-Off Finder (ECOFFinder), 97.5% subset ECOF value corresponds to the MIC value of 18.75 μg/mL. All assays were carried out in triplicate. The values presented are mean ± SEM (n = 3 independent experiments) p* and p** values are < 0.05 and < 0.001, respectively.

Although synthesized as a potential anticancer agent [5], our preliminary study showed that the novel Raja 42 isatin-benzodizole hybrid compound effectively killed several different bacterial strains including MRSA. This result led us to examine its efficacy against *C*. *difficile*. The MIC of Raja 42 against *C*. *difficile* ATCC 9689 was examined using a 96-well plate-based microdilution method as well as a disk diffusion assay on an agar plate (Fig 2A and 2B), which was determined to be 4.6 μg**/**mL (arrow in Fig 2B).

The susceptibility of *C*. *difficile* to Raja 42 was also determined with 60 different clinical isolates chosen based on a variety of criteria, including the date of isolation, the absence and presence of the toxin gene, and the antimicrobial susceptibility profile (Table 1). Because of the novelty of Raja 42, breakpoints and susceptibility parameters were determined, for which we used the ECOFF MIC breakpoint software by EUCAST. The 97.5% subset ECOFF value for Raja 42 corresponded to 18.75 μg/mL (Fig 2C). Therefore, the epidemiological susceptibility (S) cut-off corresponds to ≤ 18.75 μg/mL, and the concentration higher than 18.75 μg/mL is considered resistant (R).

**Table 1:** The effets of Raja 42 on 60 *C*. *difficile* isolates.

Sample #	Bacterial Isolates	Sampling Mon/Year	Antibiotics^a^							
			Ceftriaxone	Ciropfloxacin	Metronidazole		Vancomycin		Raja 42	
			Susceptibility	Susceptibility	MIC (μg/ml)	Susceptibility	MIC (μg/ml)	Susceptibility	MIC (μg/ml)	Susceptibility
1	CDR149	06/2012	R	R	0.75	S	0.5	S	18.75	S
2	CDR151	06/2012	R	R	0.75	S	0.75	S	4.6	S
3	CDR383	07/2012	R	R	0.25	S	0.38	S	18.75	S
4	CDR397	07/2012	R	R	0.25	S	0.38	S	18.75	S
5	CDR483	07/2012	R	R	1.5	S	1.5	S	2.3	S
6	CDR629	08/2012	R	S	0.38	S	0.75	S	37.5	R
7	CDR1071	09/2012	R	R	0.75	S	0.75	S	9.2	S
8	CDR1243	10/2012	R	S	3	R	0.75	S	18.75	S
9	CDR1305	10/2012	R	R	0.75	S	0.75	S	18.75	S
10	CDR1471	10/2012	R	R	2	S	0.5	S	9.2	S
11	CDR1625	11/2012	R	R	1	S	0.38	S	2.3	S
12	CDR1935	11/2012	R	R	3	R	0.5	S	4.6	S
13	CDR2029	12/2012	R	R	3	R	0.75	S	37.5	R
14	CDR2479	01/2013	R	R	0.5	S	0.38	S	75	R
15	CDR2903	02/2013	R	R	1	S	0.75	S	4.6	S
16	CDR3243	03/2013	R	R	0.5	S	0.5	S	4.6	S
17	CDR3501	04/2013	R	R	0.5	S	0.75	S	18.75	S
18	CDR3657	05/2013	R	R	0.75	S	0.38	S	150	R
19	CDR4031	06/2013	ND	ND	0.38	S	0.75	S	2.3	S
20	CDR4587	08/2013	R	R	1	S	1	S	75	R
21	CDR4653	09/2013	R	R	0.75	S	1.5	S	9.2	S
22	CDR4663	09/2013	R	R	0.38	S	1	S	0.55	S
23	CDR4765	12/2013	R	R	0.75	S	1.5	S	2.3	S
24	CDR4795	01/2014	ND	R	0.25	S	1	S	4.6	S
25	CDR4803	01/2014	R	R	0.38	S	1	S	4.6	S
26	CDR4829	02/2014	ND	ND	2	S	1.5	S	9.2	S
27	CDR4859	03/2014	R	R	0.5	S	1	S	2.3	S
28	CDR4867	03/2014	R	R	1	S	0.75	S	4.6	S
29	CDR4941	05/2014	R	ND	0.75	S	1	S	0.55	S
30	CDR5063	08/2014	R	R	2	S	1	S	2.3	S
31	CDR5073	08/2014	R	R	0.75	S	1.5	S	37.5	R
32	CDR5075	08/2014	R	R	8	R	1.5	S	9.2	S
33	CDR5085	09/2014	ND	R	256	R	3	R	4.6	S
34	CDR5111	10/2014	R	R	256	R	256	R	4.6	S
35	CDR5127	11/2014	R	R	0.19	S	1	S	37.5	R
36	CDR5133	11/2014	R	R	256	R	3	R	9.2	S
37	CDR5139	11/2014	R	R	256	R	3	R	4.6	S
37	CDR5179	12/2014	R	R	256	R	3	R	9.2	S
39	CDR5201	01/2015	R	ND	1	S	1.5	S	4.6	S
40	CDR5209	02/2015	R	R	0.75	S	1.5	S	150	R
41	CDR5221	02/2015	R	R	0.38	S	1.5	S	4.6	S
42	CDR5223	03/2015	R	ND	0.5	S	1.5	S	2.3	S
43	CDR5235	03/2015	R	ND	0.38	S	1.5	S	75	R
44	CDR5253	03/2015	R	R	0.38	S	1.5	S	2.3	S
45	CDR5255	04/2015	R	R	0.25	S	1.5	S	4.6	S
46	CDR5259	04/2015	R	R	2	S	1	S	4.6	S
47	CDR5269	04/2015	R	R	0.75	S	1.5	S	37.5	R
48	CDR5273	04/2015	R	R	1	S	1	S	2.3	S
49	CDR5281	04/2015	R	R	0.5	S	1.5	S	2.3	S
50	CDR5307	04/2015	R	R	0.5	S	1.5	S	4.6	S
51	CDR5313	05/2015	R	R	0.5	S	1	S	18.75	S
52	CDR5313	05/2015	R	R	6	R	1.5	S	9.2	S
53	CDR5317	05/2015	R	R	12	R	1.5	S	150	R
54	CDR5343	07/2015	R	R	1	S	1.5	S	9.2	S
55	CDR5349	07/2015	R	R	2	S	3	R	9.2	S
56	CDR5429	02/2017	R	R	0.75	S	1.5	S	2.3	S
57	CDR5441	02/2017	R	R	1.5	S	1.5	S	2.3	S
58	CDR5471	02/2017	ND	ND	3	R	1	S	1.1	S
59	CDR5473	03/2017	ND	ND	1.5	S	1	S	4.6	S
60	CDR5477	03/2017	R	R	1	S	0.75	S	4.6	S

^a^ Resistant (R) and sensitive (S) to the known antibiotics were defined according to their previously determined EUCAST susceptibility cut-off values (EUCAST, 2018). For detail of susceptibility determination to compounds, see text and legends to Figs 1 and 2.

The fact that most of the 60 clinical isolates are resistant to ceftriaxone and ciprofloxacin (Table 1) raises the possibility that these *C*. *difficile* isolate strains might have been exposed previously to β-lactam family antibacterial (like ceftriaxone) and quinolone-based compounds (like ciprofloxacin) as they are among the most frequently prescribed antimicrobials.

Since metronidazole and vancomycin are presently the two most commonly used drugs to treat patients with CDI, we examined the susceptibility of the 60 clinical isolates against these antibiotics. Among the 60 isolates, 48 (80%) and 54 (90%) were susceptible to metronidazole and vancomycin, respectively. This data indicates that metronidazole and vancomycin are quite effective for the control of *C*. *difficile* infection. However, following successful treatment of *C*. *difficile* infection with either of these antibiotics, up to 25–50% of patients experienced recurrence within a relatively short period of time [4,8–12]. Furthermore, the prolonged use of vancomycin showed several serious side effects, including nephrotoxicity [13], ototoxicity [14], and thrombocytopenia [15,16]. Therefore, it is imperative to develop effective new antibiotics that are robust against the emergence of drug resistant bacterial strains as well as having lower and fewer side effects.

The Raja 42 novel γ-lactam compound was effective against 49 of the 60 isolates. This result alone does not indicate that Raja 42 is particularly superior to metronidazole or vancomycin. It is, however, interesting to note that 10 of the 12 isolates resistant to metronidazole (CDR1243, CDR1935, CDR5075, CDR5085, CDR5111, CDR5133, CDR5139, CDR5179, CDR5313, and CDR5471) are sensitive to Raja 42. These data indicate that Raja 42 can be considered for further studies and might serve as a second-line treatment option when metronidazole is not effective. Importantly, Raja 42 effectively killed all of the five *C*. *difficile* isolates that are resistant up to 256 μg/mL of metronidazole, the maximum dose used in this experiment (and all of which are also resistant to vancomycin) (CDR5085, CDR5111, CDR5133, CDR5139, and CDR5179). Thus, our data raise the possibility that Raja 42 can be developed as an alternative drug to treat patients infected with *C*. *difficile* strains that are resistant to both metronidazole and vancomycin. It may even be possible to identify a reliable biomarker correlated to the infection of a *C*. *difficile* strain that is highly resistant to metronidazole and vancomycin. Further analysis of those five *C*. *difficile* isolates described above can lead to identifying such a biomarker. If a reliable biomarker is available, Raja 42 can be an effective first-line treatment option for certain cohort of preselected *C*. *difficile* patients.

To gain insights into the safety of Raja 42, we examined its potential therapeutic index (TI). The therapeutic index is normally calculated by comparing ED_50_ and TD_50_ values obtained from clinical trials or animal models. However, a TI value can also be estimated by comparing MIC_50_ (against bacteria) and IC_50_ (against normal human cells) values of a compound [17]. The IC_50_ of Raja 42 against 184B5 immortalized human breast cells is 93.72 μM [5], and the MIC_50_ of Raja 42 against *C*. *difficile* is 7.71 μM. These data suggest that Raja 42 TI is in the range of 12.15 (i.e., 93.72 μM/7.71 μM). This estimated value is better than that of vancomycin (TI, 7.45), suggesting that Raja 42 may be less toxic than vancomycin. However, this conclusion should be taken with caution since we have not yet examined the IC_50_ value of Raja 42 with (primary) cells from closely relevant organ sources such as intestinal tissues.

It should be noted that Raja 42 is a γ-lactam derivative. Once effectively used β-lactam-based antimicrobial compounds have substantially lost their efficacy as many bacteria have developed resistance to these antibiotics due mainly to the wide-spread use. To overcome the increasing number of resistant strains to β-lactam antibiotics, a few laboratories have tried to develop antibiotics with a γ-lactam scaffold. In these efforts, the traditional 4-membered ring of the β-lactams has been altered to a cyclopentane moiety with PBP-like activity. The first such an effort carried out was by Williams and colleagues; however, their effort was not very successful due mainly to the instability of their compounds, which was inevitably associated with poor antibacterial activity [18]. Baldwin and colleagues synthesized stable variants of γ-lactam, mainly focusing on the chemistry, but not biology [19]. Although the authors did mention in the paper that their novel compounds possessed weak antibacterial activity against gram-positive bacterial, they did not show any relevant data. Compounds based on the γ-lactam scaffold could be an effective alternative to β-lactam. However, this potential has not yet been fully explored as studies on γ-lactam thus far focussed mainly on only chemical properties [20]. Data presented here open the possibility that certain γ-lactam compounds can be highly effective against bacteria resistant to β-lactam and other antibiotics.

## Supporting information

S1 Fig (TIF)

S1 Table Includes the initial screening results of 194 *C*. *difficile* clinical samples. The 194 clinical *C*. *difficile* samples, collected from Health Sciences North (Sudbury, Ontario, Canada) by Dr. Nokehbeh and his group, are listed in S1 Table. Quality Control (QC) validation was performed comparing the MIC of our clinical isolates against that of EUCAST published values for *C*. *difficile* ATCC 700057, *C*. *difficile* ATCC 9689, *C*. *difficile* ATCC 42596, *C*. *difficile* ATCC 43598 and *E*. *faecalis* ATCC 29212 strains, respectively. All *C*. *difficile* strains were grown in Columbia broth (Oxoid Ltd., Basingstoke, Hampshire, England, Cat#: CM0331) to allow for sporulation and further growth in Brain Heart Infusion Broth (BHI) (Nutri-Bact, Terrebonne, Quebec, Canada, Cat#: QB-48-0305). Bacteriological Agar (BA) (Quelab, Montreal, Quebec, Canada, Cat: QB-46-0221) was used to prepare solid plates for MIC testing against *C*. *difficile* isolates. BA plates were supplemented with Hemin (from Bovine origins, with >90% purity) (Sigma-Aldrich, Saint Louis, MO, USA, Cat#: H9039-1G), Vitamin K1 (Sigma-Aldrich, Saint Louis, MO, USA, Cat#: V3501-1G) and laked, defibrinated sheep blood (NutriBact, Terrebonne, QC, Canada) and pre-reduced in the anaerobic chamber prior to inoculation with bacteria. *C*. *difficile* cultures were grown in anaerobic conditions in an 855-AC controlled atmospheric chamber (Plas Labs, Lansing, USA). Standard microanaerobic conditions were met (85% N_2_, 10% H_2_, 5% CO_2_). The visualization of bacteria was made using an Axioscope A1 fluorescent microscope (Carl Zeiss Microscopy, Thornwood, USA). FloQSwabs were used to create uniform and even bacterial lawns to complete an antibiotic susceptibility test. Bacterial culture density was determined using 96 well NUNC plates which were visualized in a TECAN spectra computerized plate reader at an absorbance of 540 nm.
(PDF)
