# Supplementary figures and images for: Raja 42, a novel gamma lactam compound, is effective against Clostridioides difficile
Source: PLoS One. 2021 Sep 7;16(9):e0257143. doi: 10.1371/journal.pone.0257143 (PMC8423298; doi:10.1371/journal.pone.0257143)

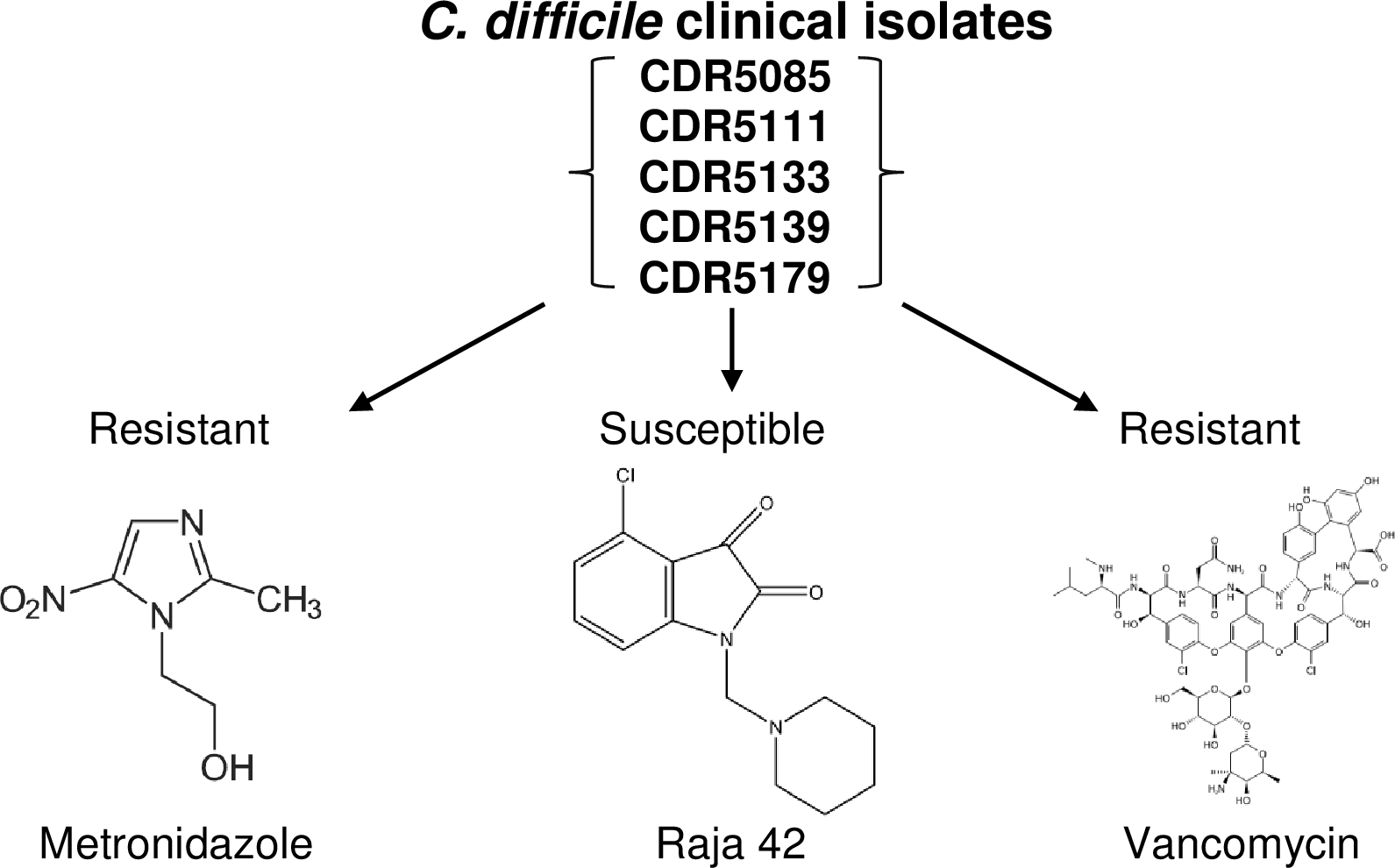

Supplement: S1 Fig — (TIF) [file pone.0257143.s001.tif]
