# Supplementary material for: Raja 42, a novel gamma lactam compound, is effective against Clostridioides difficile
Source: PLoS One. 2021 Sep 7;16(9):e0257143. doi: 10.1371/journal.pone.0257143 (PMC8423298; doi:10.1371/journal.pone.0257143)
Supplement: S1 Table — The 194 clinical C. difficile samples, collected from Health Sciences North (Sudbury, Ontario, Canada) by Dr. Nokehbeh and his group, are listed in S1 Table. Quality Control (QC) validation was performed comparing the MIC of our clinical isolates against that of EUCAST published values for C. difficile ATCC 700057, C. difficile ATCC 9689, C. difficile ATCC 42596, C. difficile ATCC 43598 and E. faecalis ATCC 29212 strains, respectively. All C. difficile strains were grown in Columbia broth (Oxoid Ltd., Basingstoke, Hampshire, England, Cat#: CM0331) to allow for sporulation and further growth in Brain Heart Infusion Broth (BHI) (Nutri-Bact, Terrebonne, Quebec, Canada, Cat#: QB-48-0305). Bacteriological Agar (BA) (Quelab, Montreal, Quebec, Canada, Cat: QB-46-0221) was used to prepare solid plates for MIC testing against C. difficile isolates. BA plates were supplemented with Hemin (from Bovine origins, with >90% purity) (Sigma-Aldrich, Saint Louis, MO, USA, Cat#: H9039-1G), Vitamin K1 (Sigma-Aldrich, Saint Louis, MO, USA, Cat#: V3501-1G) and laked, defibrinated sheep blood (NutriBact, Terrebonne, QC, Canada) and pre-reduced in the anaerobic chamber prior to inoculation with bacteria. C. difficile cultures were grown in anaerobic conditions in an 855-AC controlled atmospheric chamber (Plas Labs, Lansing, USA). Standard microanaerobic conditions were met (85% N2, 10% H2, 5% CO2). The visualization of bacteria was made using an Axioscope A1 fluorescent microscope (Carl Zeiss Microscopy, Thornwood, USA). FloQSwabs were used to create uniform and even bacterial lawns to complete an antibiotic susceptibility test. Bacterial culture density was determined using 96 well NUNC plates which were visualized in a TECAN spectra computerized plate reader at an absorbance of 540 nm. (PDF) [file pone.0257143.s002.pdf]

Supplemental S1 Table: Antibiotic susceptibility profile of 194 *C. difficile* clinical isolates.

Isolate number	Bacterial isolate[a]	Sampling time[b]	metronidazole		Vancomycin	
			MIC (µg/mL)	Susceptibility	MIC (µg/mL)	Susceptibility
Control	*C. difficile* ATCC 9689	Jun-19	2	S	2	S
Control	*C. difficile* ATCC 43596	Jun-19	2	S	2	S
Control	*C. difficile* ATCC 43598	Jun-19	1	S	2	S
Control	*C. difficile* ATCC 700057	Jun-19	0.25	S	8	S
Control	*E. faecalis* ATCC 29212	Jun-19	256	R	4	S
1	CDR27	Jun-12	1	S	1	S
2	CDR31	Jun-12	0.5	S	2	S
3	CDR149	Jun-12	1	S	0.5	S
4	CDR151	Jun-12	1	S	1	S
5	CDR159	Jun-12	1	S	1	S
6	CDR193	Jun-12	0.5	S	1	S
7	CDR203	Jun-12	1	S	2	S
8	CDR227	Jun-12	0.5	S	1	S
9	CDR273	Jul-12	0.5	S	0.5	S
10	CDR383	Jul-12	0.5	S	0.5	S
11	CDR397	Jul-12	0.5	S	0.5	S
12	CDR467	Jul-12	1	S	0.5	S
13	CDR483	Jul-12	2	S	2	S
14	CDR503	Jul-12	2	S	2	S
15	CDR567	Aug-12	1	S	1	S
16	CDR629	Aug-12	1	S	1	S
17	CDR637	Aug-12	1	S	1	S
18	CDR905	Sep-12	1	S	1	S
19	CDR911	Sep-12	1	S	1	S
20	CDR921	Sep-12	0.25	S	0.5	S
21	CDR1017	Sep-12	1	S	1	S
22	CDR1071	Sep-12	1	S	1	S
23	CDR1111	Sep-12	1	S	1	S
24	CDR1169	Sep-12	0.5	S	0.5	S
25	CDR1229	Oct-12	0.5	S	1	S
26	CDR1243	Oct-12	2	S	1	S
27	CDR1279	Oct-12	1	S	2	S
28	CDR1305	Oct-12	1	S	1	S
29	CDR1341	Oct-12	1	S	1	S
30	CDR1377	Oct-12	0.5	S	1	S
31	CDR1467	Oct-12	0.5	S	0.5	S
32	CDR1471	Oct-12	2	S	1	S
33	CDR1625	Nov-12	1	S	0.5	S

34	CDR1891	Nov-12	1	S	1	S
35	CDR1903	Nov-12	1	S	1	S
36	CDR1935	Nov-12	1	S	1	S
37	CDR2029	Dec-12	1	S	1	S
38	CDR2479	Jan-13	0.5	S	0.5	S
39	CDR2523	Jan-13	0.5	S	0.5	S
40	CDR2903	Feb-13	0.5	S	1	S
41	CDR2915	Feb-13	0.5	S	1	S
42	CDR3175	Mar-13	1	S	0.75	S
43	CDR3243	Mar-13	0.5	S	0.5	S
44	CDR3501	Apr-13	0.5	S	1	S
45	CDR3601	Apr-13	0.5	S	1	S
46	CDR3657	May-13	1	S	0.5	S
47	CDR3861	May-13	1	S	1	S
48	CDR4031	Jun-13	1	S	2	S
49	CDR4175	Jun-13	1	S	4	R
50	CDR4257	Jul-13	1	S	0.5	S
51	CDR4365	Jul-13	0.5	S	1	S
52	CDR4373	Jul-13	0.5	S	1	S
53	CDR4465	Jul-13	0.5	S	1	S
54	CDR4513	Aug-13	0.5	S	2	S
55	CDR4545	Aug-13	0.5	S	1	S
56	CDR4587	Aug-13	1	S	1	S
57	CDR4653	Sep-13	1	S	2	S
58	CDR4663	Sep-13	0.5	S	1	S
59	CDR4669	Sep-13	1	S	1	S
60	CDR4681	Sep-13	0.5	S	1	S
61	CDR4687	Oct-13	1	S	1	S
62	CDR4703	Oct-13	0.5	S	1	S
63	CDR4707	Oct-13	0.5	S	1	S
64	CDR4719	Nov-13	0.5	S	1	S
65	CDR4725	Nov-13	0.5	S	1	S
66	CDR4739	Nov-13	0.5	S	1	S
67	CDR4741	Nov-13	0.5	S	2	S
68	CDR4753	Dec-13	0.5	S	1	S
69	CDR4761	Dec-13	0.5	S	1	S
70	CDR4765	Dec-13	1	S	2	S
71	CDR4771	Dec-13	0.5	S	1	S
72	CDR4795	Jan-14	0.5	S	1	S
73	CDR4803	Jan-14	0.5	S	1	S
74	CDR4811	Jan-14	0.5	S	1	S
75	CDR4813	Jan-14	0.5	S	1	S

76	CDR4819	Feb-14	0.5	S	2	S
77	CDR4821	Feb-14	0.25	S	1	S
78	CDR4825	Feb-14	0.5	S	2	S
79	CDR4829	Feb-14	2	S	2	S
80	CDR4839	Feb-14	1	S	1	S
81	CDR4847	Feb-14	1	S	2	S
82	CDR4851	Feb-14	1	S	1	S
83	CDR4859	Mar-14	0.5	S	1	S
84	CDR4867	Mar-14	1	S	1	S
85	CDR4881	Mar-14	1	S	1	S
86	CDR4897	Apr-14	2	S	2	S
87	CDR4903	Apr-14	1	S	2	S
88	CDR4909	Apr-14	1	S	1	S
89	CDR4911	Apr-14	1	S	2	S
90	CDR4917	Apr-14	0.5	S	1	S
91	CDR4921	Apr-14	0.5	S	1	S
92	CDR4941	May-14	1	S	1	S
93	CDR4949	May-14	0.5	S	1	S
94	CDR4967	May-14	0.5	S	1	S
95	CDR4983	Jun-14	0.5	S	1	S
96	CDR5015	Jun-14	0.5	S	2	S
97	CDR5029	Jun-14	0.5	S	1	S
98	CDR5041	Jul-14	0.5	S	2	S
99	CDR5057	Jul-14	4	R	2	S
100	CDR5059	Jul-14	2	S	2	S
101	CDR5063	Aug-14	2	S	1	S
102	CDR5065	Aug-14	1	S	2	S
103	CDR5067	Aug-14	1	S	1	S
104	CDR5071	Aug-14	2	S	1	S
105	CDR5073	Aug-14	1	S	2	S
106	CDR5075	Aug-14	12	R	2	S
107	CDR5085	Sep-14	256	R	4	R
108	CDR5093	Sep-14	256	R	2	S
109	CDR5099	Sep-14	1	S	1	S
110	CDR5107	Oct-14	0.5	S	2	S
111	CDR5111	Oct-14	256	R	256	R
112	CDR5117	Oct-14	0.5	S	1	S
113	CDR5121	Nov-14	0.25	S	1	S
114	CDR5127	Nov-14	256	R	4	R
115	CDR5133	Nov-14	256	R	4	R
116	CDR5137	Nov-14	1	S	2	S
117	CDR5139	Dec-14	256	R	4	R

118	CDR5141	Dec-14	8	R	1	S
119	CDR5145	Dec-14	0.5	S	2	S
120	CDR5153	Dec-14	0.5	S	1	S
121	CDR5155	Dec-14	0.5	S	1	S
122	CDR5159	Jan-15	0.5	S	2	S
123	CDR5161	Jan-15	0.5	S	1	S
124	CDR5163	Jan-15	0.5	S	1	S
125	CDR5165	Jan-15	0.5	S	2	S
126	CDR5167	Jan-15	0.5	S	1	S
127	CDR5171	Jan-15	0.5	S	2	S
128	CDR5179	Jan-15	1	S	2	S
129	CDR5181	Jan-15	1	S	2	S
130	CDR5183	Jan-15	1	S	2	S
131	CDR5185	Jan-15	0.5	S	2	S
132	CDR5189	Jan-15	1	S	2	S
133	CDR5201	Feb-15	1	S	2	S
134	CDR5209	Feb-15	0.5	S	2	S
135	CDR5211	Feb-15	1	S	1	S
136	CDR5213	Feb-15	1	S	1	S
137	CDR5221	Mar-15	0.5	S	2	S
138	CDR5223	Mar-15	0.5	S	2	S
139	CDR5225	Mar-15	0.5	S	1	S
140	CDR5229	Mar-15	0.5	S	2	S
141	CDR5233	Mar-15	1	S	2	S
142	CDR5235	Mar-15	0.5	S	2	S
143	CDR5237	Mar-15	0.5	S	2	S
144	CDR5239	Mar-15	0.5	S	1	S
145	CDR5243	Mar-15	1	S	2	S
146	CDR5245	Mar-15	1	S	2	S
147	CDR5247	Mar-15	1	S	1	S
148	CDR5249	Mar-15	0.5	S	1	S
149	CDR5253	Apr-15	0.5	S	2	S
150	CDR5255	Apr-15	2	S	1	S
151	CDR5259	Apr-15	1	S	2	S
152	CDR5267	Apr-15	0.5	S	2	S
153	CDR5269	Apr-15	1	S	1	S
154	CDR5273	Apr-15	0.5	S	2	S
155	CDR5277	Apr-15	1	S	2	S
156	CDR5281	Apr-15	0.5	S	2	S
157	CDR5287	May-15	0.5	S	1	S
158	CDR5291	May-15	1	S	2	S
159	CDR5293	May-15	0.5	S	2	S

160	CDR5297	May-15	0.5	S	2	S
161	CDR5299	May-15	0.5	S	1	S
162	CDR5305	May-15	0.5	S	2	S
163	CDR5307	May-15	0.5	S	1	S
164	CDR5309	May-15	0.5	S	2	S
165	CDR5313	May-15	8	R	2	S
166	CDR5317	May-15	16	R	2	S
167	CDR5319	May-15	1	S	2	S
168	CDR5321	May-15	1	S	4	R
169	CDR5329	Jun-15	2	S	2	S
170	CDR5331	Jun-15	1	S	2	S
171	CDR5335	Jun-15	2	S	1	S
172	CDR5343	Jul-15	1	S	2	S
173	CDR5345	Jul-15	0.5	S	1	S
174	CDR5347	Jul-15	1	S	2	S
175	CDR5349	Jul-15	2	S	4	R
176	CDR5351	Jul-15	1	S	2	S
177	CDR5429	Feb-17	1	S	2	S
178	CDR5431	Feb-17	1	S	2	S
179	CDR5433	Feb-17	1	S	2	S
180	CDR5435	Feb-17	0.5	S	1	S
181	CDR5439	Feb-17	2	S	2	S
182	CDR5441	Feb-17	2	S	2	S
183	CDR5445	Feb-17	2	S	2	S
184	CDR5447	Feb-17	2	S	4	R
185	CDR5449	Feb-17	1	S	2	S
186	CDR5451	Jan-17	2	S	2	S
187	CDR5461	Jan-17	2	S	2	S
188	CDR5463	Jan-17	1	S	2	S
189	CDR5465	Feb-17	2	S	2	S
190	CDR5469	Feb-17	2	S	1	S
191	CDR5471	Feb-17	4	R	1	S
192	CDR5473	Mar-17	2	S	1	S
193	CDR5475	Mar-17	2	S	2	S
194	CDR5477	Mar-17	1	S	1	S

[a] The isolates designated with CDR followed by a number are clinical isolates from patient stools diagnosed with CDI at Health Sciences North hospital, Sudbury, Ontario, Canada.
[b] The sampling time the controls (rows 1-5) are the time of purchase from ATCC. Other numbers show isolation time at the Health Sciences North in month and year.
